# Medical Image Fusion Based on Sparse Representation and PCNN in NSCT Domain

**DOI:** 10.1155/2018/2806047

**Published:** 2018-05-24

**Authors:** Jingming Xia, Yiming Chen, Aiyue Chen, Yicai Chen

**Affiliations:** ^1^School of Electronics and Information Engineering, Nanjing University of Information Science and Technology, Nanjing 210044, China; ^2^School of Mechanical Engineering, North China Electric Power University, Hebei 071000, China

## Abstract

The clinical assistant diagnosis has a high requirement for the visual effect of medical images. However, the low frequency subband coefficients obtained by the NSCT decomposition are not sparse, which is not conducive to maintaining the details of the source image. To solve these problems, a medical image fusion algorithm combined with sparse representation and pulse coupling neural network is proposed. First, the source image is decomposed into low and high frequency subband coefficients by NSCT transform. Secondly, the K singular value decomposition (K-SVD) method is used to train the low frequency subband coefficients to get the overcomplete dictionary *D*, and the orthogonal matching pursuit (OMP) algorithm is used to sparse the low frequency subband coefficients to complete the fusion of the low frequency subband sparse coefficients. Then, the pulse coupling neural network (PCNN) is excited by the spatial frequency of the high frequency subband coefficients, and the fusion coefficients of the high frequency subband coefficients are selected according to the number of ignition times. Finally, the fusion medical image is reconstructed by NSCT inverter. The experimental results and analysis show that the algorithm of gray and color image fusion is about 34% and 10% higher than the contrast algorithm in the edge information transfer factor QAB/F index, and the performance of the fusion result is better than the existing algorithm.

## 1. Introduction

Medical imaging attracts more and more attention due to the increasing requirements of clinic investigation and disease diagnosis [1]. Imaging of different modalities can reflect different information about the lesion. For example, CT images have a clear bone image, but soft tissue imaging is blurred; MRI images can obtain multiangle and multiplane detail information of soft tissue, but the skeleton imaging is blurred; PET images can present metabolic activity of human cells, but the anatomical structure is not clear. Therefore, the medical images of different modalities are fused to improve the accuracy and recognition of lesion location, which provides a more effective imaging reference for clinical diagnosis of modern medicine [2].

Compared with wavelet transform and Contourlet transform, NSCT transform has the advantages of multiscale and multidirection analysis, and anisotropy and translation invariance [3]. However, the low frequency subband coefficients obtained by NSCT decomposition are not sparse, and the fusion of them directly is not conducive to maintain the characteristics of the source image. Sparse Representation (SR) can extract deeper structural features between low frequency subband coefficients and express or approximate it in a linear combination of a few atoms [4]. Compared with other artificial neural networks, PCNN has an incomparable advantage over other traditional artificial neural networks [5]. The PCNN model has global coupling and pulse synchronization, which can combine the input high frequency subband coefficients with human visual characteristics to obtain richer detail information [6]. Therefore, NSCT transform, sparse representation, and PCNN model image fusion method are gaining more and more attention. Chun-hui and Yun-ting [7] propose a fast image fusion algorithm based on sparse representation and nonsubsampled Contourlet transform. This algorithm greatly improves the efficiency of image fusion, but it gives only four-directional sparse representation in the low frequency subband, which cannot fully represent the characteristics and details of the source image. Shabanzade and Ghassemian [8] propose a multimodal image fusion algorithm based on NSCT and sparse representation. This algorithm uses sparse representation to perfectly approximate the low frequency subband coefficients. However, the rule based on the larger local energy or variance is used for high frequency subband coefficients, which cannot effectively solve the problem of image detail smoothing caused by sparse representation. Gong et al. [9] propose an image fusion method based on the improved NSCT transform and PCNN model, and this method can preserve the image structure better so that the fusion image is more in line with the human visual nervous system. However, the mutual information of the fusion image is relatively less. Mohammed et al. [10] propose a medical image fusion algorithm based on sparse representation and dual input PCNN model. This algorithm has a high fusion performance and adapts to the human visual nerve system. However, it needs to train medical image database to get an overcomplete dictionary; in addition, PCNN model applies a dual input, which presents the high complexity and low integration efficiency of the algorithm.

In order to obtain the medical fusion image with high fusion performance and high fusion efficiency, and to help it adapt to human visual nervous system, this paper, by aiming at the above research situation and existing problems and combining the sparse representation with PCNN simplified model, proposes the medical image fusion algorithm based on NSCT and SR-PCNN, hereinafter referred to as NSCT-SR-PCNN fusion algorithm.

## 2. Nonsubsampled Contourlet Transform

**Figure 1:**
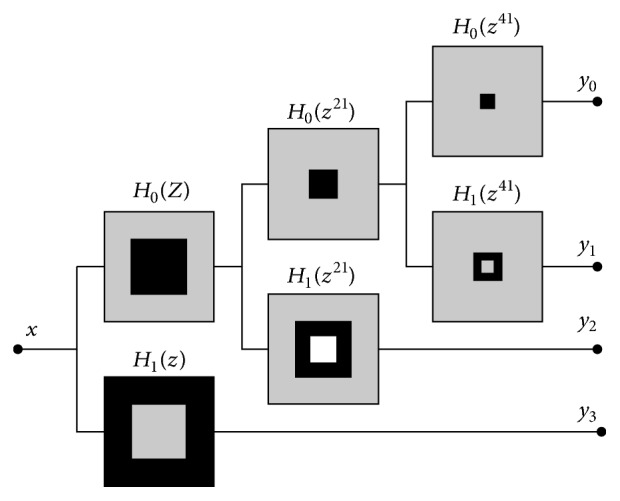
NSPFB filter decomposition.

The NSCT transform consists of two steps: the nondownsampling pyramid (NSP) decomposition and the nondownsampling direction filter bank (NSDFB). NSP decomposition is the process of decomposing the source image into low and high frequency subbands through the nonsubsampling tower filter bank to ensure the characteristic of NSCT multiscale transformation. The NSPFB filter is decomposed as shown in Figure 1.

**Figure 2:**
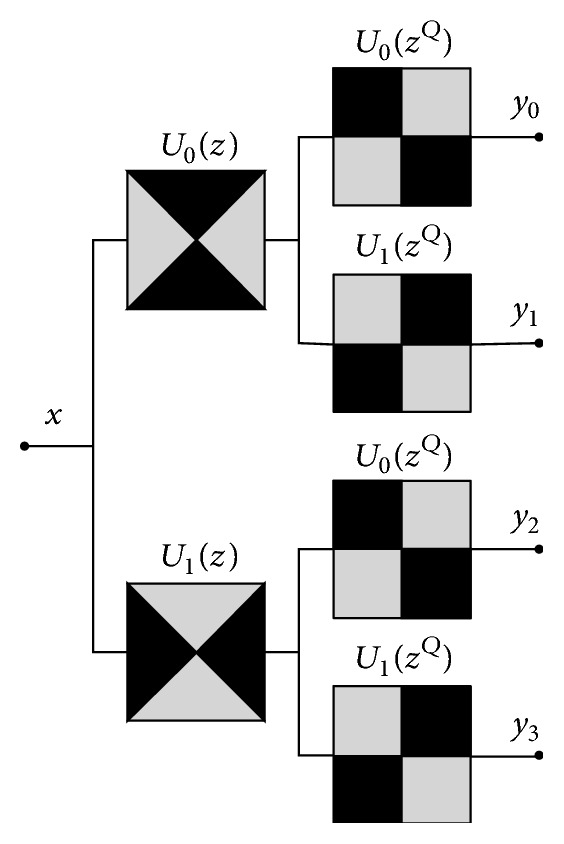
NSDFB filter decomposition.

NSDFB is a two-channel nondownsampling filter bank, and it decomposes the high frequency subband image decomposed by NSP in level-one NSDFB direction, so it can produce 2^l^ different directional subband image. NSDFB filter is decomposed as shown in Figure 2.

## 3. Sparse Representation

Sparse representation means that the natural signal can be represented or approximated by a linear combination of a small number of atoms in the overcomplete dictionary *D* ∈ *R*^*n*×*k*^; then the sparse coefficient of the signal *x* can be obtained by

(1) minA A0,s.t. X−DA22<ε,

where *D* is a prespecified dictionary; *A* is a sparse coefficient vector; ‖*A*‖_0_ stands for the count of nonzero entries in *A*; *ε* is the bounded representation error. NSCT-SR-PCNN algorithm uses K-SVD method to train the dictionary and uses the orthogonal matching tracking optimization (OMP) algorithm to estimate the sparse coefficient *A* [11].

## 4. Pulse Coupled Neural Network

**Figure 3:**
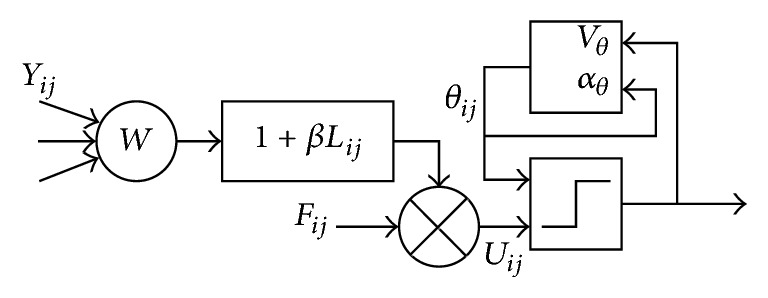
PCNN simplified model.

PCNN simplified model is a feedback neural network model proposed by simulating the signal processing mechanism of cat visual cortex [12]. In the simplified model, the partial simplification of the parameters makes the generality of the model well guaranteed. However, there is a great difference in the response of the visual system to the different feature regions in the image. In the PCNN model, this difference is mainly reflected in the setting of the parameters, and the flexible changes in the parameters still affect the final fusion results. Therefore, this paper uses the most commonly used discrete mathematical iterative model. The simplified model is shown in Figure 3.

The mathematical expression of the PCNN simplified model can be expressed by

(2) Fijn=IijLijn=exp⁡−αLLijn−1+VL∑k,lWijklYijn−1Uijn=Fijn1+βLijnθijn=exp⁡−αθθijn−1+VθYijn−1Yijn−1=1,Uijn>θijn0,Uijn≤θijn,

where *n* is the number of iterations; *I*_*ij*_ is the external input; *Y*_*ij*_ is the output of neuron; *U*_*ij*_ is the internal behavior of neurons; *F*_*ij*_ is feedback input excitation; *L*_*ij*_ is the input of neuron's link; *W*_*ijkl*_ is the weight coefficient of the connection between neurons; *β* is the link strength coefficient; *θ*_*ij*_ is the output of variable threshold function; *V*_*L*_ and *α*_*L*_ are, respectively, the signal amplification factor and attenuation time constant of neuron's link; *V*_*θ*_ and *α*_*θ*_ are, respectively, the signal amplification factor and decay time constant of variable threshold function.

## 5. Medical Image Fusion Algorithm Based on NSCT-SR-PCNN

**Figure 4:**
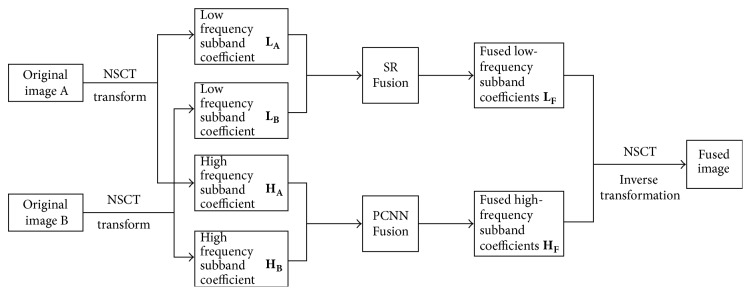
NSCT-SR-PCNN medical image fusion algorithm flow.

NSCT-SR-PCNN algorithm firstly uses NSCT transform to decompose the source image after registration to obtain the low frequency and high frequency subband of the source image; secondly the fusion method based on sparse representation is used to fuse the low frequency subband, and the fusion method based on PCNN simplified model is used to fuse the high frequency subband; finally NSCT inverse transform is used to reconstruct the fused subband coefficients to obtain the medical image of fusion. The specific implementation process of NSCT-SR-PCNN medical image fusion algorithm is shown in Figure 4.

### 5.1. The Rules of Low Frequency Subband Coefficient Fusion

Low frequency subband coefficient fusion is achieved by using sparse representation fusion. First of all, blocks taken from the image to be fused form a training sample set, and secondly K-SVD algorithm is used to train a complete dictionary, and then the Batch-OMP [13] optimization algorithm is used to estimate the sparse coefficient; finally, the sparse coefficients are adaptively fused according to image features. The specific steps are as follows.

**Step 1 .**

Use the NSCT transforms to decompose, respectively, the source images *A* and *B* with *M* × *N* size after registration to obtain the low frequency and high frequency subband coefficients.

**Step 2 .**

Segment the low frequency subband coefficients *L*_*A*_ and *L*_*B*_ by using the sliding window with the steps of *S* pixels and the size *n* × *n*, and obtain the (*N* + *n* − 1)×(*M* + *n* − 1) subblocks; transform the image subblocks into column vectors to form the sample training matrix *V*_*A*_ and *V*_*B*_.

**Step 3 .**

Average the sample training matrices *V*_*A*_ and *V*_*B*_ to obtain the mean matrices $\overset{\wedge }{V_{A}}$ and $\overset{\wedge }{V_{B}}$; average of the sample training matrices *V*_*A*_ and *V*_*B*_ is removed to obtain the sparse representation of the sample matrices *V*_*A*_′ and *V*_*B*_′.

**Step 4 .**

Use the K-SVD algorithm to iterate the sample matrix to obtain the overcomplete dictionary matrix *D* of the low frequency subband coefficient.

**Step 5 .**

Use the Batch-OMP optimization algorithm to estimate the sparse coefficients of *V*_*A*_′ and *V*_*B*_′ and obtain the sparse coefficient matrices *α*_*A*_ and *α*_*B*_. According to the value of *L*_1_ norm, the sparse coefficient matrix of column *i* is fused by applying the rules of

(3) $$\begin{array}{c} \overset{i}{\alpha_{F}}\;\;=\left\{\begin{array}{cc} \overset{i}{\alpha_{A}}+\frac{1}{2}\overset{i}{\alpha_{B}}, & \text{if }\overset{n}{\underset{k=1}{\sum }}{\left\|\overset{k}{\alpha_{A}}\right\|}_{1}>\begin{array}{c} \overset{n}{\underset{k=1}{\sum }}{\left\|\overset{k}{\alpha_{B}}\right\|}_{1},\;\;\overset{i}{\alpha_{A}}\;<\;\overset{i}{\alpha_{B}},\;\overset{i}{\alpha_{A}}\cdot \overset{i}{\alpha_{B}}\;<0 \end{array}\\ \overset{i}{\alpha_{B}}+\frac{1}{2}\overset{i}{\alpha_{A}}, & \text{if }\overset{n}{\underset{k=1}{\sum }}{\left\|\overset{k}{\alpha_{A}}\right\|}_{1}<\begin{array}{c} \overset{n}{\underset{k=1}{\sum }}{\left\|\overset{k}{\alpha_{B}}\right\|}_{1},\;\;\overset{i}{\alpha_{A}}\;>\;\overset{i}{\alpha_{B}},\;\overset{i}{\alpha_{A}}\cdot \overset{i}{\alpha_{B}}\;<0 \end{array}\\ \frac{\overset{i}{\alpha_{A}}+\overset{i}{\alpha_{B}}}{2}+\frac{1}{2}\overset{i}{\alpha_{A}}, & \text{if }\overset{n}{\underset{k=1}{\sum }}{\left\|\overset{k}{\alpha_{A}}\right\|}_{1}=\overset{n}{\underset{k=1}{\sum }}{\left\|\overset{k}{\alpha_{B}}\right\|}_{1},\;\;\overset{i}{\alpha_{A}}\;>\;\overset{i}{\alpha_{B}},\;\overset{i}{\alpha_{A}}\cdot \overset{i}{\alpha_{B}}\;<0\\ \frac{\overset{i}{\alpha_{A}}+\overset{i}{\alpha_{B}}}{2}+\frac{1}{2}\overset{i}{\alpha_{B}}, & \text{if }\overset{n}{\underset{k=1}{\sum }}{\left\|\overset{k}{\alpha_{A}}\right\|}_{1}=\begin{array}{c} \overset{n}{\underset{k=1}{\sum }}{\left\|\overset{k}{\alpha_{B}}\right\|}_{1},\;\;\overset{i}{\alpha_{A}}\;<\;\overset{i}{\alpha_{B}},\;\overset{i}{\alpha_{A}}\cdot \overset{i}{\alpha_{B}}\;<0 \end{array}\\ \alpha^{F}, & \text{otherwise} \end{array}\right. \end{array}$$

Among them, *α*^*F*^ can be seen from

(4) $$\begin{array}{c} \alpha^{F}=\left\{\begin{array}{cc} \overset{i}{\alpha_{A}}, & \text{if }\overset{n}{\underset{k=1}{\sum }}{\left\|\overset{k}{\alpha_{A}}\right\|}_{1}\begin{array}{c} >\overset{n}{\underset{k=1}{\sum }}{\left\|\overset{k}{\alpha_{B}}\right\|}_{1} \end{array}\\ \overset{i}{\alpha_{B}}, & \text{if }\overset{n}{\underset{k=1}{\sum }}{\left\|\overset{k}{\alpha_{A}}\right\|}_{1}\begin{array}{c} <\overset{n}{\underset{k=1}{\sum }}{\left\|\overset{k}{\alpha_{B}}\right\|}_{1} \end{array}\\ \frac{\overset{i}{\alpha_{A}}+\overset{i}{\alpha_{B}}}{2}, & \text{if }\overset{n}{\underset{k=1}{\sum }}{\left\|\overset{k}{\alpha_{A}}\right\|}_{1}\begin{array}{c} =\overset{n}{\underset{k=1}{\sum }}{\left\|\overset{k}{\alpha_{B}}\right\|}_{1} \end{array}. \end{array}\right. \end{array}$$

**Step 6 .**

The choice of the fusion mean matrix is given by

(5) $$\begin{array}{c} \overset{\wedge }{V_{F}}\;\;=\left\{\begin{array}{cc} \overset{\wedge }{V_{A}}, & \text{if }\overset{n}{\underset{k=1}{\sum }}{\left\|\overset{k}{\alpha_{A}}\right\|}_{1}\begin{array}{c} >\overset{n}{\underset{k=1}{\sum }}{\left\|\overset{k}{\alpha_{B}}\right\|}_{1} \end{array}\\ \overset{\wedge }{V_{B}}, & \text{if }\overset{n}{\underset{k=1}{\sum }}{\left\|\overset{k}{\alpha_{A}}\right\|}_{1}\begin{array}{c} <\overset{n}{\underset{k=1}{\sum }}{\left\|\overset{k}{\alpha_{B}}\right\|}_{1} \end{array}\\ \frac{\overset{\wedge }{V_{A}}+\overset{\wedge }{V_{B}}}{2}, & \text{if }\overset{n}{\underset{k=1}{\sum }}{\left\|\overset{k}{\alpha_{A}}\right\|}_{1}\begin{array}{c} =\overset{n}{\underset{k=1}{\sum }}{\left\|\overset{k}{\alpha_{B}}\right\|}_{1} \end{array}. \end{array}\right. \end{array}$$

**Step 7 .**

Multiply the overcomplete dictionary matrix *D* with the fusion sparse coefficient matrix *α*^*F*^ and then add fusion mean matrix $\overset{\wedge }{V_{F}}$. The fusion sample training matrix *V*_*F*_ is given by

(6) $$\begin{array}{c} V_{F}=D\alpha_{F}\;+\overset{\wedge }{V_{F}}. \end{array}$$

**Step 8 .**

Convert the columns of the fusion sample training matrix *V*_*F*_ into data subblocks, and reconstruct the data subblocks to obtain the fusion coefficients of the low frequency subbands.

**Figure 5:**
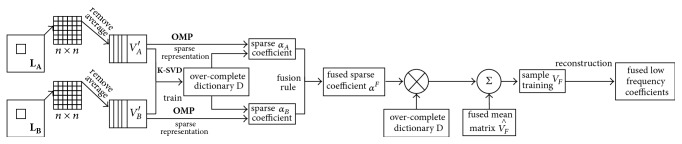
Low frequency subband coefficient SR fusion process.

The implementation of low frequency subband coefficients fusion based on sparse representation is shown in Figure 5.

### 5.2. The Rules of High Frequency Subband Coefficient Fusion

According to the characteristics of human visual system, the spatial frequency (SF) reflects the local area characteristics and details of the image. The high frequency subband coefficient fusion selects SF as the neuron feedback input to stimulate the PCNN simplified model. The neuron feedback input is expressed by

(7) $$\begin{array}{c} F_{ij}={SF}_{ij}=\sqrt{R{F_{ij}}^{2}+C{F_{ij}}^{2}} \end{array}$$

Among them are the window size 3 × 3, *RF*_*ij*_, and *CF*_*ij*_; from formula (8) we can see:

(8) RFij=1M×N∑i=1M ∑j=2NXi,j−Xi,j−12CFij=1M×N∑i=2M ∑j=1NXi,j−Xi−1,j2

In the PCNN model, the value of *β* determines the strength of the coupling relationship of the neurons, and the high frequency subband coefficient fusion selects the Laplacian energy (EOL), the visibility (VI), and the standard deviation (SD) that can measure the neighborhood characteristic information, respectively, as the linking strength values of PCNN corresponding neurons, and EOL, VI, and SD are expressed by

(9) EOL=∑u,v∈wfuu+fvv2VI=1N∑u,v∈w1mkα · fu,v−mkmkSD=∑i=1l∑j=1lfu,v−mk2l∙l,

where (*u*, *v*) is a pixel point of the image; *f*(*u*, *v*) is the pixel value; *w* is the window of size *l* × *l*; *m*_*k*_ is the pixel gray level average; *N* is the number of pixels in the window; *α* is a constant.

For fusion based on PCNN simplified model, SF is used as the neuron feedback input to excite each neuron, and EOL, VI, and SD are selected as the linking strength values of the corresponding neurons; then the corresponding ignition map is obtained by the PCNN ignition, and the new ignition map of the source image is constructed by the weighting function. Finally, the fusion coefficient is selected according to the number of ignition frequencies. Specific implementation steps are as follows.

**Step 9 .**

According to formula (7), calculate the neighborhood spatial frequency SF_*A*_ and SF_*B*_ of the high frequency subband coefficients HA and HB and then normalize SF_*A*_ and SF_*B*_, and mark them as SF_*A*_′ and SF_*B*_′, respectively; SF_*A*_′ and SF_*B*_′ are used as neuron feedback input to motivate the PCNN simplified model.

**Step 10 .**

According to formula (9), calculate EOL, VI, and SD of high frequency subband coefficients HA and HB (which is recorded as *β*_*AE*_, *β*_*AV*_, *β*_*AS*_, *β*_*BE*_, *β*_*BV*_, and *β*_*BS*_) and take them, respectively, as the linking strength value of corresponding neurons.

**Step 11 (initialization setting).**

*L*_*ij*_(0) = *U*_*ij*_(0) = 0, *θ*_*ij*_(0) = 1; at this time the neuron is in the flameout state; that is, *Y*_*ij*_(0) = 0, so the number of pulses generated is *O*_*ij*_(0) = 0.

**Step 12 .**

According to formula (2), calculate *L*_*ij*_[*n*], *U*_*ij*_[*n*], *θ*_*ij*_[*n*], and *Y*_*ij*_[*n*].

**Step 13 .**

The output of the PCNN simplified model iteration run is as follows: *O*_*AE*_, *O*_*AV*_, *O*_*AS*_, *O*_*BE*_, *O*_*BV*_, and *O*_*BS*_; use the weighting function to obtain the new ignition map *O*_*A*_ and *O*_*B*_ which corresponds to high frequency subband coefficients HA and HB: *O*_*A*_ = *w*_1_*O*_*AE*_ + *w*_2_*O*_*AV*_ + *w*_3_*O*_*AS*_, *O*_*B*_ = *w*_4_*O*_*BE*_ + *w*_5_*O*_*BV*_ + *w*_6_*O*_*BS*_; *w*_*i*_  (*i* = 1,2, 3,4, 5,6) is given by

(10) w1=OAEOAE+OAV+OASw2=OAVOAE+OAV+OASw3=OASOAE+OAV+OASw4=OBEOBE+OBV+OBSw5=OBVOBE+OBV+OBSw6=OBSOBE+OBV+OBS.

**Step 14 .**

Compare the ignition time output threshold values (ignition frequencies) at the new ignition map pixel; the high frequency subband fusion coefficient *H*_*F*_(*i*, *j*) is given by

(11) HFi,j=HAi,j,if  OAi,j>OBi,jHBi,j,if  OAi,j<OBi,jHAi,j+HBi,j2,if  OAi,j=OBi,j.

**Figure 6:**
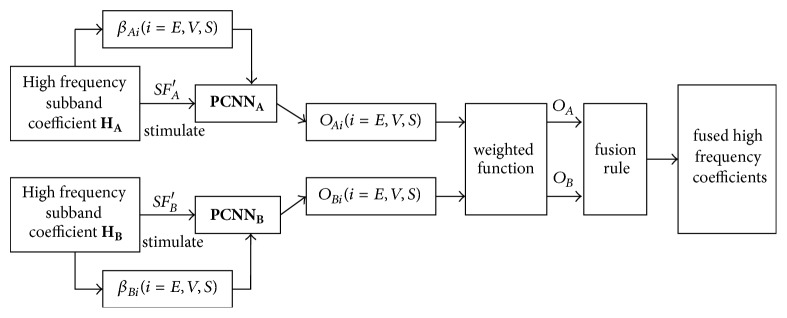
High frequency subband coefficient PCNN fusion process.

The adaptive fusion implementation process based on PCNN simplified model is shown in Figure 6.

## 6. The Results and Analysis of Experiments

In order to verify the effectiveness of the proposed algorithm, five kinds of contrast algorithms are selected to conduct gray and color medical image fusion experiments. The medical images of each group are obtained from http://www.med.harvard.edu/AANLIB/home.html page. Objective evaluation of quality is made in terms of 7 indexes, such as the information entropy (IE), spatial frequency (SF), mean gradient (AG) [14], clarity (MC), mutual information (MI), standard deviation (SD) [15], and edge information delivery factor (*Q*^AB/F^ high weight evaluation index) [16–19]. The visual information fidelity (VIFF) and structural similarity model (SSIM) were used to evaluate the visual effect of human eyes. The contrast algorithm 1 is a medical image fusion study based on NSCT transform (referred to as NSCT fusion algorithm) proposed in the paper [20]. The contrast algorithm 2 is the multifocus image fusion (referred to as the SR fusion algorithm) based on the fragmented complete sparse representation proposed in the paper [21]. The contrast algorithm 3 is the image fusion by using pulse coupled neural network (referred to as PCNN fusion algorithm), proposed by the paper [22]. The contrast algorithm 4 is a multifocus image fusion based on NSCT and sparse representation (referred to as NSCT-SR fusion algorithm) proposed in the paper [11]. The contrast algorithm 5 is an improved algorithm based on NSCT and adaptive PCNN medical image fusion (referred to as NSCT-PCNN fusion algorithm) proposed in the paper [23]. NSCT transformation parameters setting: the class of decomposition is 4, the scale decomposition filter selects “pyrexc” filter, and the direction filter selects “vk” filter [24]; SR parameters setting: sliding window size is 8 × 8, step size is 1, the number of dictionary training iteration is 30 times, and sparse error is 0.01; PCNN parameters setting: *V*_*L*_ = 1, *V*_*θ*_ = 20, *α*_*L*_ = 1, *α*_*θ*_ = 0.2, *N*_max_ = 100, and

(12) $$\begin{array}{c} w=\left[\begin{array}{ccc} 0.707 & 1 & 0.707\\ 1 & 0 & 1\\ 0.707 & 1 & 0.707 \end{array}\right] \end{array}$$

(see [25]).

### 6.1. Gray Image Fusion Experiment

**Figure 7:**
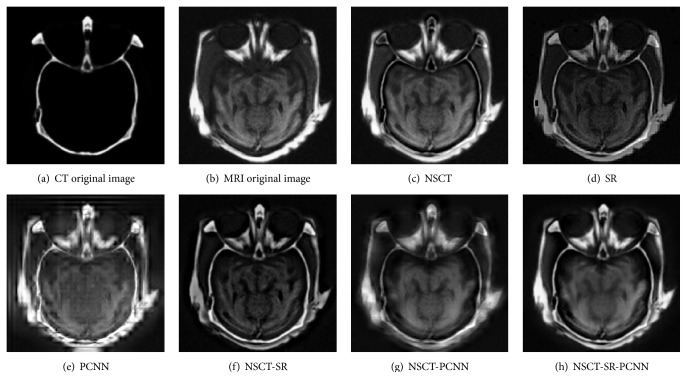
CT/MRI medical image fusion results.

**Figure 8:**
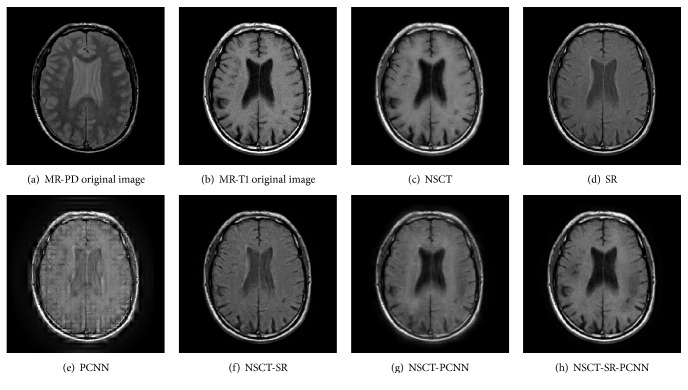
MR-PD/MR-T1 medical image fusion results.

**Figure 9:**
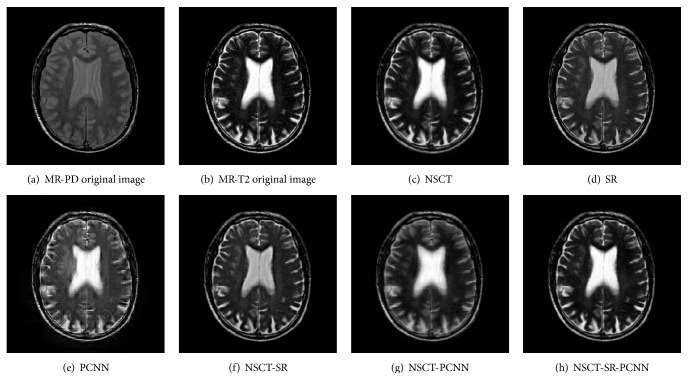
MR-PD/MR-T2 medical image fusion results.

**Figure 10:**
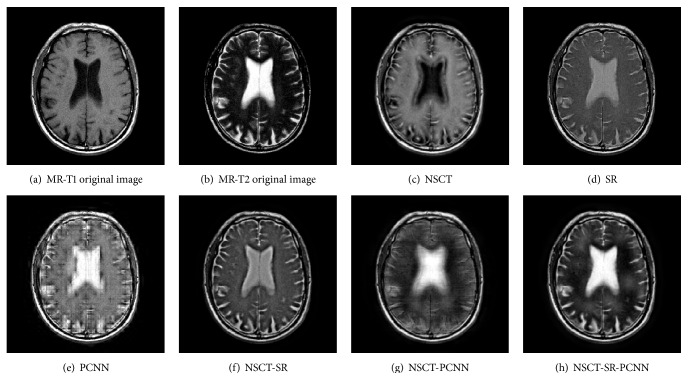
MR-T1/MR-T2 medical image fusion results.

Gray image fusion experiment is conducted by selecting the images of four groups of brain under different states as the images to be fused. The results of the fusion of the various algorithms are shown in Figures 7–10, and the objective evaluation indexes for quality of the various algorithms are shown in Tables 1–4.

The NSCT-SR-PCNN algorithm has better fusion and better fusion performance than the five contrast algorithms, from the human visual effects in Figures 7–10 or the evaluation index from Tables 1–4. The reason is that, for NSCT algorithm, NSCT decomposition of the low frequency subband coefficient is not sparse, and direct low frequency coefficient fusion is not conducive to the retention of the source image features; for SR algorithm and the PCNN algorithm, the image fusion is based on the spatial domain implementation, and the spatial domain fusion method fails to express details, so the fusion image has low contrast, fuzzy details, and block artifact, and other problems. For NSCT-SR algorithm, it solves the problem that low frequency subband coefficient is not sparse, but the fusion of high frequency subband is only conducted based on the direction featured principle, which cannot completely present the details of the image information. For NSCT-PCNN algorithm, it can adapt to the human visual system, but the low frequency subband coefficients have no sparseness. For NSCT-SR-PCNN algorithm, it not only solves the problem of the detail loss of the wavelet transform and the sparseness loss of low frequency subband coefficient of the NSCT, but also improves the comprehensive performance of the fusion results by using the spatial frequency of the high frequency subband coefficients to impel input and by using EOL, VI, and SD to strengthen their links with the corresponding neurons. NSCT-SR-PCNN algorithm can obtain better performance for CT/MRI, MR-PD/MR-T1 and MR-T1/MR-T2 medical image fusion in the comprehensive analysis of evaluation indexes. The NSCT-SR-PCNN algorithm can achieve better performance for CT/MRI, MR-PD/MR-T1 and MR-PD/MR-T2 medical image fusion according to edge information delivery factor of *Q*^AB/F^ index.

### 6.2. Color Image Fusion Experiment

**Figure 11:**
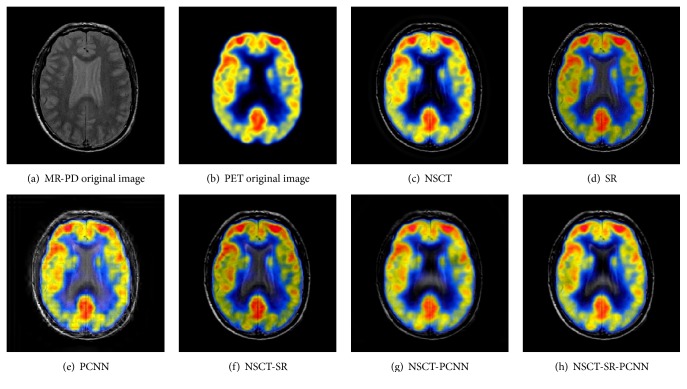
MR-PD/PET medical image fusion results.

**Figure 12:**
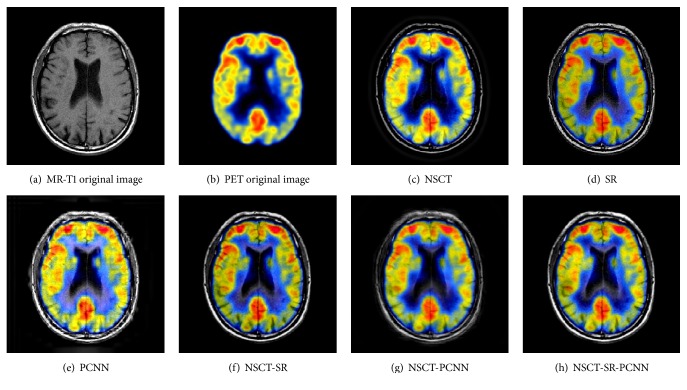
MR-T1/PET medical image fusion results.

**Figure 13:**
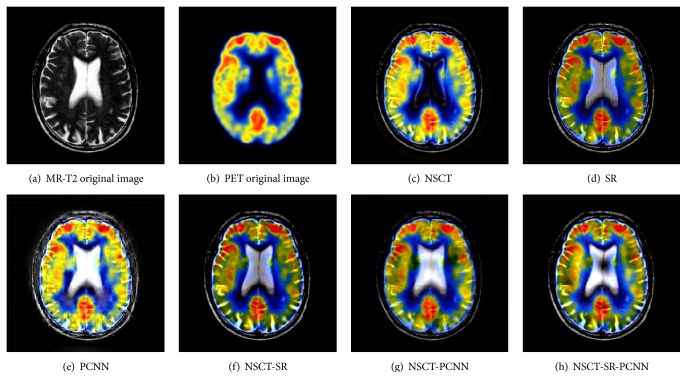
MR-T2/PET medical image fusion results.

Color image fusion experiment selects three groups of brain under different images as the images to be fused. The fusion results of various algorithms are shown in Figures 11–13, and the objective evaluation index for quality of various algorithms is shown in Tables 5–7.

Compared with the five contrast algorithms in terms of the human visual effect of Figures 11–13 or the comprehensive analysis of the evaluation index and the *Q*^AB/F^ index of the edge information delivery factor, the NSCT-SR-PCNN algorithm can provide better performance for MR-PD/PET, MR-T1/PET, and MR-T2/PET medical image fusion. Based on the experimental data, the NSCT-SR-PCNN algorithm proposed in this paper can make the fusion image obtain high fusion performance in the aspect of texture clarity, gray scale variation, and contrast ratio and can realize no color loss or distortion in transmission.

## 7. Conclusion

The NSCT-SR-PCNN algorithm effectively combines NSCT transform, sparse representation, and pulse coupled neural network to overcome the shortcomings of wavelet transform, which cannot reflect the holistic characteristics, and solves the problem that the low frequency subband coefficient is not sparse. In addition, this algorithm collects the image texture, the degree of change in edge and details, and other information, which improves the comprehensive performance of the fusion results. The experimental data show that although not all the evaluation indexes of NSCT-SF-PCNN algorithm rank the first, the evaluation indexes of the NSCT-SF-PCNN algorithm are all in the top three and the top four; besides this, the comprehensive indexes are the number one, and the edge information delivery factor *Q*_AB/F_ of the high weight evaluation index is higher than that of the five contrast algorithms, and the edge and detail information of the source image is better preserved, and the human visual effect is better. Certainly, the NSCT-SR-PCNN algorithm also needs to be improved. For example, through the online dictionary learning method, to obtain a complete dictionary *D* still requires further study.

## Figures and Tables

**Table 1:** Quality evaluation of CT/MRI medical image fusion.

Evaluation index	NSCT	SR	PCNN	NSCT-SR	NSCT-PCNN	NSCT-SR-PCNN
IE	0.1312	**0.1498**	0.1000	**0.3965**	0.0620	**0.1634**
MC	**2.6621**	2.3780	**2.9962**	2.3718	2.4343	**2.4396**
MI	**3.4885**	**2.8146**	1.8082	2.1005	1.7618	**2.2426**
SD	**56.6060**	37.9078	**59.5349**	44.0044	46.2033	**55.4061**
*Q* ^AB/F^	**0.6572**	0.5441	0.4020	**0.6794**	0.3502	**0.6899**
VIFF	0.6598	0.5688	0.3922	**0.7074**	0.4201	**0.6677**
SSIM	**0.9385**	0.8775	0.6674	0.8520	0.7896	**0.9567**

**Table 2:** Quality evaluation of MR-PD/MR-T1 medical image fusion.

Evaluation index	NSCT	SR	PCNN	NSCT-SR	NSCT-PCNN	NSCT-SR-PCNN
IE	**0.9999**	0.9904	0.9579	0.9922	**0.9999**	**0.9963**
MC	2.2233	2.4200	**4.3005**	2.4234	**2.7476**	**2.6054**
MI	**3.0047**	**2.4785**	2.1328	2.3858	2.2826	**2.5474**
SD	**54.6893**	45.1990	**56.0878**	45.4664	46.5353	**51.4444**
*Q* ^AB/F^	**0.5648**	**0.5669**	0.4345	0.5399	0.4205	**0.5887**
VIFF	0.5722	0.5675	0.3408	0.4964	0.4228	**0.5991 **
SSIM	1.4165	**1.5332**	0.9745	1.4869	1.3444	**1.4279**

**Table 3:** Quality evaluation of MR-PD/MR-T2 medical image fusion.

Evaluation index	NSCT	SR	PCNN	NSCT-SR	NSCT-PCNN	NSCT-SR-PCNN
IE	0.9730	**0.9768**	**0.9928**	**0.9777**	**0.9859**	**0.9768**
MC	**2.5614**	**2.5205**	**3.0647**	2.4164	2.4802	**2.5033**
MI	2.3808	**2.7229**	2.3961	**2.5019**	**2.4154**	**2.5378**
SD	**56.4522**	46.3235	**59.0546**	47.5818	**52.1596**	**56.6857**
*Q* ^AB/F^	**0.6120**	**0.6491**	0.4912	**0.6385**	0.4612	**0.6530**
VIFF	0.7091	0.7528	0.5534	0.6966	0.6267	** 0.8150 **
SSIM	1.6297	1.6524	1.5168	1.6552	1.6306	**1.7148**

**Table 4:** Quality evaluation of MR-T1/MR-T2 medical image fusion.

Evaluation index	NSCT	SR	PCNN	NSCT-SR	NSCT-PCNN	NSCT-SR-PCNN
IE	0.9936	0.9907	**0.9979**	0.9920	**0.9974**	**0.9956**
MC	2.7955	2.6224	**3.2850**	2.6689	**2.8966**	**2.8338**
MI	1.8604	**2.4779**	2.1623	**2.2494**	2.0621	**2.2047**
SD	53.9478	47.4264	**72.9638**	49.5285	**54.9129**	**58.7637**
*Q* ^AB/F^	0.4321	**0.5076**	0.4357	**0.5754**	0.4007	**0.5732**
VIFF	0.3003	0.4529	0.3316	0.3400	0.3400	**0.4769 **
SSIM	1.2341	** 1.3590**	1.2475	1.2379	1.2379	**1.2812**

**Table 5:** Quality evaluation of MR-PD/PET medical image fusion.

Evaluation index	NSCT	SR	PCNN	NSCT-SR	NSCT-PCNN	NSCT-SR-PCNN
IE	**0.9977**	0.9761	0.9677	0.9791	**0.9954**	**0.9801**
SF	**6.0266**	5.7013	**6.9747**	5.8537	5.7295	**5.9952**
AG	4.8934	**4.9109**	**6.9312**	4.8133	4.2310	**4.9744**
MC	**1.9402**	1.7960	**2.5601**	1.8627	1.8753	**1.9409**
MI	**2.7632**	**2.7705**	2.6597	2.6677	2.6979	**2.7879**
SD	**70.2719**	50.0841	**70.9987**	50.8343	63.5030	**67.2682**
*Q* ^AB/F^	**0.5414**	**0.5411**	0.4311	0.5295	0.3908	**0.5718**

**Table 6:** Quality evaluation of MR-T1/PET medical image fusion.

Evaluation index	NSCT	SR	PCNN	NSCT-SR	NSCT-PCNN	NSCT-SR-PCNN
IE	**0.9992**	0.9879	0.9755	0.9897	**0.9975**	**0.9923**
SF	6.6819	**6.9951**	**7.9528**	6.9423	6.7408	**6.9475**
AG	6.5924	**8.0276**	**9.9589**	7.5923	6.2566	**7.5961**
MC	2.2740	2.3869	**3.0047**	2.3844	**2.4070**	**2.4067**
MI	2.5493	**2.7738**	2.5631	**2.7455**	2.5485	**2.7559**
SD	**70.8814**	60.3674	**77.6841**	62.4699	63.5939	**67.0245**
*Q* ^AB/F^	0.4333	**0.5505**	0.4521	**0.5611**	0.3908	**0.5681**

**Table 7:** Quality evaluation of MR-T2/PET medical image fusion.

Evaluation index	NSCT	SR	PCNN	NSCT-SR	NSCT-PCNN	NSCT-SR-PCNN
IE	**0.9953**	0.9697	0.9622	0.9734	**0.9935**	**0.9769**
SF	6.7434	**6.9677**	**8.0595**	6.6804	6.7986	**6.9365**
AG	6.7453	**8.0305**	**10.3970**	7.1794	6.0200	**7.4678**
MC	2.2195	**2.2513**	**3.0121**	2.1432	2.2898	**2.3136**
MI	2.5588	**2.7566**	2.5835	**2.6558**	**2.6199**	2.6076
SD	**70.6653**	54.3247	**82.1519**	56.6147	63.2384	**65.1174**
*Q* ^AB/F^	0.3985	**0.5125**	0.3832	**0.5449**	0.3166	**0.5451**

## References

[1] Fei Y., Wei G., Zongxi S. (2017). Medical image fusion based on feature extraction and sparse representation. *International Journal of Biomedical Imaging*.

[2] Zhen-yi J., Yuan-jun W. (2016). Multi-modality medical image fusion method based on non-subsampled contourlet transform. *Chinese Journal of Medical Physics*.

[3] Do M. N., Vetterli M. Contourlets: A directional multiresolution image representation.

[4] Zhao-yu S., Rong H., Ning O. (2015). Image fusion based on multi-scale sparse representation. *Computer Engineering and Design*.

[5] Eckhorn R., Reitboeck H. J., Arndt M. (1989). A neural network for feature linking via synchronous activity. *Canadian Journal of Microbiology*.

[6] Reitboeck H. J., Eckhorn R., Arndt M., Dicke P. (1990). A Model for Feature Linking via Correlated Neural Activity. *Synergetics of Cognition*.

[7] Chun-hui Z., Yun-ting G. (2016). Fast image fusion algorithm based on sparse representation and non-subsampled contourlet transform. *Journal of Electronics and Information Technology*.

[8] Shabanzade F., Ghassemian H. Multimodal image fusion via sparse representation and clustering-based dictionary learning algorithm in NonSubsampled Contourlet domain.

[9] Gong J., Wang B., Qiao L., Xu J., Zhang Z. Image Fusion Method Based on Improved NSCT Transform and PCNN Model.

[10] Mohammed A., Nisha K. L., Sathidevi P. S. A novel medical image fusion scheme employing sparse representation and dual PCNN in the NSCT domain.

[11] Ning O., Xue-ying Z., Hua Y. (2017). Multi-focus image fusion based on NSCT and sparse representation. *Computer Engineering and Design*.

[12] Gray C. M., Singer W. (1987). Stimulus specific neuronal oscillations in the cat visual cortex: A cortical functional unit. *Soc.neurosci.abst*.

[13] Hai-feng Z., Yu-miao L., Ming L., Si-bao C. (2014). Medical Image Compression Based on Fast Sparse Representation. *Computer Engineering*.

[14] Yuan-jun W., Bo-yu J., Zhen-yi J. (2013). Review of multimodal medical image fusion technology based on wavelet transformation. *Chinese Journal of Medical Physics*.

[15] Wei-liang X., Wen-zhan D., Jun-feng L. (2016). Medical image fusion algorithm based on lifting wavelet transform and PCNN. *Journal of Zhejiang Sci-Tech University*.

[16] Xydeas C. S., Petrović V. (2000). Objective image fusion performance measure. *IET Journals and Magazines on Electronics Letters*.

[17] Qu G., Zhang D., Yan P. (2002). Information measure for performance of image fusion. *IET Journals and Magazines on Electronics Letters*.

[18] Piella G., Heijmans H. A new quality metric for image fusion.

[19] Liu Z., Blasch E., Xue Z., Zhao J., Laganiére R., Wu W. (2012). Objective assessment of multiresolution image fusion algorithms for context enhancement in Night vision: A comparative study. *IEEE Transactions on Pattern Analysis and Machine Intelligence*.

[20] Xiu-hua T., Wang X. (2013). Research on NSCT-based Medical Image Fusion. *Computer Applications and Software*.

[21] Yao-jia C., Yong-ping Z., Jian-yan T. (2012). Multi-focus Image Fusion Based on Blocked Sparse Representation. *Video Engineering*.

[22] Hao C., Juan Z., Yan-ying L. (2010). Image fusion based on pulse coupled neural network. *Optics and Precision Enginee Ring*.

[23] Jun-qiang C., Dan-fei H. (2015). A Medical Image Fusion Improved Algorithm Based on NSCT and Adaptive PCNN. *Journal of Changchun University of Science and Technology*.

[24] Li S., Yang B., Hu J. (2011). Performance comparison of different multi-resolution transforms for image fusion. *Information Fusion*.

[25] Tian Y., Li Y., Ye F. Multimodal medical image fusion based on nonsubsampled contourlet transform using improved PCNN.

